# (2,9-Dimethyl-1,10-phenanthroline-κ^2^ *N*,*N*′)bis­(2-hydroxy­benzoato-κ*O*)copper(II)

**DOI:** 10.1107/S1600536808036283

**Published:** 2008-11-13

**Authors:** Cuiping Zhai, Feng-mei Yan, Pei-zheng Zhao

**Affiliations:** a College of Chemistry and Chemical Engineering, Henan University, Kaifeng 475001, People’s Republic of China; b Department of Chemistry and Chemical Engineering, Huanghuai University, Zhumadian 463000, People’s Republic of China; c College of Chemistry and Environmental Science, Henan Normal University, Xinxiang 453007, People’s Republic of China

## Abstract

The Cu^II^ atoms in the two independent mol­ecules of the title compound, [Cu(C_7_H_5_O_3_)_2_(C_14_H_12_N_2_)], are each coordinated by a bidentate 2,9-dimethyl-1,10-phenanthroline (dmphen) mol­ecule and two monodentate 2-hydroxy­benzoate anions in a distorted tetra­hedral geometry. The crystal packing is stabilized by intra­molecular hydrogen bonding and π–π inter­actions between the dmphen rings of neighboring mol­ecules, with distances between their ring planes of 3.5670 (4) and 3.5181 (9) Å.

## Related literature

For the features of metal–phenanthroline complexes, see: Naing *et al.* (1995 ▶); Wang *et al.* (1996 ▶); Wall *et al.* (1999 ▶). For related structures, see: Cheng *et al.* (2007 ▶); Xuan *et al.* (2007 ▶); Zhao *et al.* (2007 ▶).
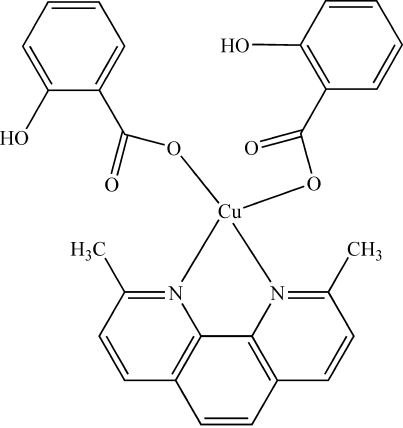


## Experimental

### 

#### Crystal data

[Cu(C_7_H_5_O_3_)_2_(C_14_H_12_N_2_)]
*M* *_r_* = 546.02
Monoclinic, 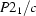

*a* = 23.819 (2) Å
*b* = 12.2576 (11) Å
*c* = 17.9084 (17) Å
β = 112.023 (1)°
*V* = 4847.0 (8) Å^3^
*Z* = 8
Mo *K*α radiation
μ = 0.95 mm^−1^
*T* = 291 (2) K
0.30 × 0.21 × 0.19 mm

#### Data collection

Bruker APEXII CCD area-detector diffractometer
Absorption correction: multi-scan (*SADABS*; Sheldrick, 2004 ▶) *T*_min_ = 0.765, *T*_max_ = 0.837
30637 measured reflections
8932 independent reflections
5274 reflections with *I* > 2σ(*I*)
*R*_int_ = 0.060

#### Refinement

*R*[*F* ^2^ > 2σ(*F* ^2^)] = 0.045
*wR*(*F* ^2^) = 0.121
*S* = 1.01
8932 reflections
675 parameters
H-atom parameters constrained
Δρ_max_ = 0.38 e Å^−3^
Δρ_min_ = −0.45 e Å^−3^

### 

Data collection: *APEX2* (Bruker, 2004 ▶); cell refinement: *APEX2* and *SAINT* (Bruker, 2004 ▶); data reduction: *SAINT*; program(s) used to solve structure: *SHELXS97* (Sheldrick, 2008 ▶); program(s) used to refine structure: *SHELXL97* (Sheldrick, 2008 ▶); molecular graphics: *SHELXTL* (Sheldrick, 2008 ▶); software used to prepare material for publication: *publCIF* (Westrip, 2008 ▶).

## Supplementary Material

Crystal structure: contains datablocks I, global. DOI: 10.1107/S1600536808036283/hg2438sup1.cif

Structure factors: contains datablocks I. DOI: 10.1107/S1600536808036283/hg2438Isup2.hkl

Additional supplementary materials:  crystallographic information; 3D view; checkCIF report

## Figures and Tables

**Table:** 

Cu1—O2	1.931 (2)
Cu1—O4	1.946 (2)
Cu1—N2	1.994 (3)
Cu1—N1	2.022 (3)
Cu2—O10	1.945 (2)
Cu2—O8	1.956 (3)
Cu2—N4	1.992 (3)
Cu2—N3	2.044 (3)

**Table:** 

O2—Cu1—O4	91.00 (11)
O2—Cu1—N2	152.67 (12)
O4—Cu1—N2	97.15 (11)
O2—Cu1—N1	104.90 (11)
O4—Cu1—N1	144.27 (11)
N2—Cu1—N1	83.30 (11)
O10—Cu2—O8	90.72 (11)
O10—Cu2—N4	94.59 (12)
O8—Cu2—N4	155.88 (12)
O10—Cu2—N3	143.61 (12)
O8—Cu2—N3	106.20 (12)
N4—Cu2—N3	82.98 (13)

**Table 2:** Hydrogen-bond geometry (Å, °)

*D*—H⋯*A*	*D*—H	H⋯*A*	*D*⋯*A*	*D*—H⋯*A*
O12—H12⋯O11	0.82	1.82	2.549 (4)	147
O9—H9⋯O7	0.82	1.84	2.561 (4)	146
O6—H6⋯O5	0.82	1.85	2.572 (4)	146
O3—H3⋯O1	0.82	1.82	2.553 (5)	148

**Fig. 1.**
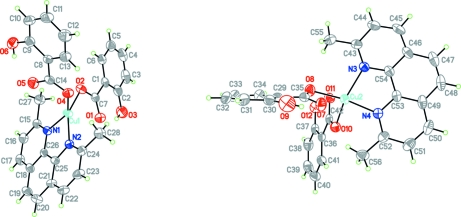
The molecular structure of the title complex(I), with atom labels and 30% probability displacement ellipsoids.

**Fig. 2.**
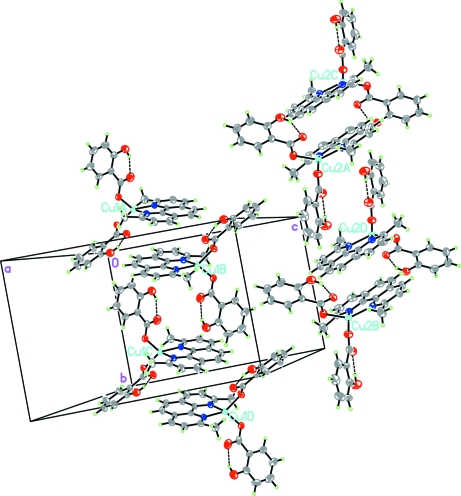
π—π interactions of neighboring molecules and intramolecular hydrogen bonds in the crystal structure of (I).[symmetry code: (Cu1A) x, y - 1, z; (Cu1B) -x + 1, -y + 1, -z + 1; (Cu1D) -x + 1, -y + 2, -z + 1; (Cu2A) -x + 2, y - 1/2, -z + 3/2; (Cu2B) -x + 2, y + 1/2, -z + 3/2; (Cu2C) x, -y - 1/2, z + 3/2; (Cu2D) x, -y + 1/2, z + 3/2]

## supplementary crystallographic information

### Comment

Metal-phenanthroline complexes have attracted much attention because of their peculiar features (Wang *et al.*, 1996; Wall *et al.*, 1999; Naing *et al.*, 1995). Some Cu(II)-phenanthroline complexes have been synthesized and structures were determined (Cheng *et al*., 2007; Xuan *et al.*, 2007; Zhao *et al.*, 2007). Recently, we obtained the title compound copper(II) complex (I), by reaction of 2,9-dimethyl-1,10-phenanthroline, 2-hydroxybenzoic acid and Cu(NO_3_)_2_ in an ethanol/water mixture. The structure of the title compound, Cu(C_14_H_12_N_2_)(C_6_H_4_OHCOO)_2_,(I), is shown below.

There are two independent molecules in the asymmetric unit. Each Cu^II^ ion is four-coordinated by two N atoms from a 2,9-dimethyl-1,10-phenanthroline ligand, and two O atoms from two 2-hydroxybenzoic anions. The Cu^II^ ion locates in the center, and CuO_2_N_2_ unit forms a distorted tetrahedral geometry (Fig.1). The Cu—N and Cu—O bond lengths in two independent molecules different slightly (Table 1). The hydroxy directions of 2-hydroxybenzoic anions in the two independent molecules are also different.

An intramolecular hydrogen bond between the hydroxy group and uncoordinated carboxyl O atom stabilizes the conformation of the hydroxybenzoate ligands (Table 2). A partially overlapped arrangement of neighboring parallel Cu1A-dmphen [symmetry code: (Cu1A) *x*, *y* - 1, *z*] and Cu1B-dmphen rings [symmetry code: (Cu1B) -*x* + 1, -*y* + 1, -*z* + 1], Cu2A-dmphen [symmetry code: (Cu2A) -*x* + 2, *y* - 1/2, -*z* + 3/2] and Cu2C-dmphen rings [symmetry code: (Cu2C) *x*, -*y* - 1/2, *z* + 3/2] are observed in the structure of (I) (Fig.2). The shorter face-to-face separation of 3.5670 (4)Å and 3.5181 (9)Å indicates the existence of π—π stacking between the dmphen ligands.

### Experimental

2-hydroxybenzoic acid (0.1389 g, 1 mmol) and NaOH (0.0370 g, 1 mmol) were dissolved in distilled water(10 ml) and Cu(NO_3_)_2_.3H_2_O (0.1222 g, 0.5 mmol) were added. This solution was added to a solution of 2,9-dimethyl-1,10-phenanthroline hemihydrate (C_14_H_12_N_2_.0.5H_2_O, 0.1090 g, 0.5 mmol) in ethanol (10 ml). The mixture was stirred at 323 K and then refluxed for 5 h, cooled to room temperature and filtered. Green single crystals of (I) were appeared over a period of eighteen days by slow evaporation at room temperature.

### Refinement

Methyl H and hydroxy H atoms were placed in calculated positions,with C—H=0.96 and O—H=0.82 Å, and refined with free torsion angles to fit the electron density; *U*_iso_(H) = 1.5*U*_eq_(carrier). Other H atoms were placed in calculated positions, with C—H=0.93 Å, and refined in the riding-model approximation with *U*_iso_(H) = 1.2*U*_eq_(C).

### Crystal data

**Table:**

[Cu(C_7_H_5_O_3_)_2_(C_14_H_12_N_2_)]	*F*_000_ = 2248
*M**_r_* = 546.02	*D*_x_ = 1.496 Mg m^−^^3^
Monoclinic, *P*2_1_/*c*	Mo *K*α radiation λ = 0.71073 Å
Hall symbol: -P 2ybc	Cell parameters from 3270 reflections
*a* = 23.819 (2) Å	θ = 2.4–19.2º
*b* = 12.2576 (11) Å	µ = 0.95 mm^−^^1^
*c* = 17.9084 (17) Å	*T* = 291 (2) K
β = 112.023 (1)º	Block, green
*V* = 4847.0 (8) Å^3^	0.30 × 0.21 × 0.19 mm
*Z* = 8	

### Data collection

**Table:**

Bruker APEXII CCD area-detector diffractometer	8932 independent reflections
Radiation source: fine-focus sealed tube	5274 reflections with *I* > 2σ(*I*)
Monochromator: graphite	*R*_int_ = 0.060
*T* = 291(2) K	θ_max_ = 25.5º
φ and ω scans	θ_min_ = 2.4º
Absorption correction: multi-scan(SADABS; Sheldrick, 2004)	*h* = −28→27
*T*_min_ = 0.765, *T*_max_ = 0.837	*k* = −14→14
30637 measured reflections	*l* = −21→21

### Refinement

**Table:**

Refinement on *F*^2^	Secondary atom site location: difference Fourier map
Least-squares matrix: full	Hydrogen site location: inferred from neighbouring sites
*R*[*F*^2^ > 2σ(*F*^2^)] = 0.045	H-atom parameters constrained
*wR*(*F*^2^) = 0.122	*w* = 1/[σ^2^(*F*_o_^2^) + (0.051*P*)^2^ + 0.2481*P*] where *P* = (*F*_o_^2^ + 2*F*_c_^2^)/3
*S* = 1.01	(Δ/σ)_max_ = 0.001
8932 reflections	Δρ_max_ = 0.38 e Å^−^^3^
675 parameters	Δρ_min_ = −0.45 e Å^−^^3^
Primary atom site location: structure-invariant direct methods	Extinction correction: none

### Special details

**Table:**

Geometry. All e.s.d.'s (except the e.s.d. in the dihedral angle between two l.s. planes)are estimated using the full covariance matrix. The cell e.s.d.'s are takeninto account individually in the estimation of e.s.d.'s in distances, anglesand torsion angles; correlations between e.s.d.'s in cell parameters are onlyused when they are defined by crystal symmetry. An approximate (isotropic)treatment of cell e.s.d.'s is used for estimating e.s.d.'s involving l.s. planes.
Refinement. Refinement of *F*^2^ against ALL reflections. The weighted *R*-factor *wR* andgoodness of fit *S* are based on *F*^2^, conventional *R*-factors *R* are basedon *F*, with *F* set to zero for negative *F*^2^. The threshold expression of*F*^2^ > σ(*F*^2^) is used only for calculating *R*-factors(gt) *etc*. and isnot relevant to the choice of reflections for refinement. *R*-factors basedon *F*^2^ are statistically about twice as large as those based on *F*, and *R*-factors based on ALL data will be even larger.

### Fractional atomic coordinates and isotropic or equivalent isotropic displacement parameters (Å^2^)

**Table:**

	*x*	*y*	*z*	*U*_iso_*/*U*_eq_	
Cu1	0.416752 (19)	0.76816 (4)	0.34347 (3)	0.04372 (14)	
Cu2	0.91833 (2)	0.24372 (4)	0.06488 (3)	0.04679 (15)	
O1	0.42604 (14)	0.5478 (3)	0.36158 (18)	0.0828 (10)	
O2	0.37117 (12)	0.6601 (2)	0.26599 (15)	0.0537 (7)	
O3	0.4003 (2)	0.3447 (3)	0.3513 (2)	0.1039 (13)	
H3	0.4177	0.4014	0.3714	0.156*	
O4	0.42450 (12)	0.8580 (2)	0.25824 (15)	0.0525 (7)	
O5	0.33691 (13)	0.9202 (2)	0.25654 (16)	0.0636 (8)	
O6	0.26673 (13)	1.0250 (3)	0.13213 (18)	0.0715 (8)	
H6	0.2774	0.9978	0.1772	0.107*	
O7	0.92602 (13)	0.4499 (2)	0.07908 (17)	0.0690 (8)	
O8	0.85069 (12)	0.3372 (2)	0.06067 (17)	0.0611 (7)	
O9	0.90651 (19)	0.6548 (3)	0.0833 (3)	0.1015 (12)	
H9	0.9264	0.5995	0.0853	0.152*	
O10	0.92554 (11)	0.1888 (2)	0.16999 (15)	0.0540 (7)	
O11	0.84406 (12)	0.0976 (2)	0.09313 (15)	0.0568 (7)	
O12	0.79075 (13)	−0.0325 (2)	0.15603 (18)	0.0675 (8)	
H12	0.7973	−0.0009	0.1198	0.101*	
N1	0.38290 (13)	0.7629 (2)	0.43145 (16)	0.0390 (7)	
N2	0.49111 (13)	0.8211 (2)	0.43227 (17)	0.0404 (7)	
N3	0.90189 (14)	0.1985 (2)	−0.05127 (18)	0.0461 (8)	
N4	1.00369 (13)	0.2094 (2)	0.08003 (19)	0.0456 (8)	
C1	0.35133 (17)	0.4709 (3)	0.2453 (2)	0.0479 (10)	
C2	0.3600 (2)	0.3653 (4)	0.2763 (3)	0.0668 (12)	
C3	0.3263 (3)	0.2798 (4)	0.2294 (4)	0.0814 (16)	
H3A	0.3305	0.2098	0.2509	0.098*	
C4	0.2874 (2)	0.2979 (4)	0.1529 (4)	0.0787 (15)	
H4	0.2660	0.2397	0.1220	0.094*	
C5	0.27904 (19)	0.4010 (4)	0.1202 (3)	0.0681 (12)	
H5	0.2524	0.4125	0.0675	0.082*	
C6	0.31061 (17)	0.4865 (3)	0.1665 (2)	0.0543 (10)	
H6A	0.3047	0.5564	0.1448	0.065*	
C7	0.38553 (18)	0.5648 (4)	0.2941 (2)	0.0521 (10)	
C8	0.36927 (17)	0.9750 (3)	0.1521 (2)	0.0444 (9)	
C9	0.3138 (2)	1.0240 (3)	0.1071 (2)	0.0552 (11)	
C10	0.3058 (2)	1.0737 (4)	0.0339 (3)	0.0713 (13)	
H10	0.2688	1.1055	0.0037	0.086*	
C11	0.3519 (3)	1.0761 (4)	0.0061 (3)	0.0842 (16)	
H11	0.3459	1.1088	−0.0431	0.101*	
C12	0.4071 (2)	1.0306 (4)	0.0503 (3)	0.0806 (14)	
H12A	0.4386	1.0337	0.0317	0.097*	
C13	0.4154 (2)	0.9797 (3)	0.1232 (2)	0.0606 (11)	
H13	0.4527	0.9483	0.1529	0.073*	
C14	0.37703 (19)	0.9146 (3)	0.2272 (2)	0.0474 (10)	
C15	0.32818 (16)	0.7343 (3)	0.4286 (2)	0.0445 (9)	
C16	0.31365 (18)	0.7396 (3)	0.4971 (2)	0.0535 (10)	
H16	0.2753	0.7183	0.4938	0.064*	
C17	0.35443 (19)	0.7751 (3)	0.5680 (3)	0.0545 (11)	
H17	0.3446	0.7770	0.6136	0.065*	
C18	0.41203 (17)	0.8093 (3)	0.5725 (2)	0.0452 (9)	
C19	0.45829 (19)	0.8514 (3)	0.6437 (2)	0.0568 (11)	
H19	0.4510	0.8555	0.6912	0.068*	
C20	0.5121 (2)	0.8852 (3)	0.6438 (2)	0.0603 (11)	
H20	0.5408	0.9140	0.6905	0.072*	
C21	0.52535 (16)	0.8772 (3)	0.5721 (2)	0.0467 (10)	
C22	0.58051 (18)	0.9085 (3)	0.5682 (2)	0.0587 (11)	
H22	0.6104	0.9403	0.6125	0.070*	
C23	0.59019 (17)	0.8921 (3)	0.4987 (2)	0.0579 (11)	
H23	0.6272	0.9118	0.4962	0.069*	
C24	0.54513 (16)	0.8458 (3)	0.4307 (2)	0.0479 (10)	
C25	0.48185 (16)	0.8339 (3)	0.5027 (2)	0.0389 (8)	
C26	0.42421 (16)	0.8003 (3)	0.5022 (2)	0.0374 (8)	
C27	0.28159 (17)	0.6985 (4)	0.3492 (2)	0.0632 (12)	
H27A	0.2888	0.7348	0.3061	0.095*	
H27B	0.2419	0.7169	0.3473	0.095*	
H27C	0.2843	0.6210	0.3435	0.095*	
C28	0.55725 (18)	0.8220 (4)	0.3559 (2)	0.0657 (12)	
H28A	0.5362	0.7567	0.3311	0.099*	
H28B	0.6000	0.8122	0.3698	0.099*	
H28C	0.5433	0.8819	0.3190	0.099*	
C29	0.83703 (17)	0.5201 (3)	0.0916 (2)	0.0480 (10)	
C30	0.8550 (2)	0.6281 (4)	0.0941 (2)	0.0621 (12)	
C31	0.8204 (3)	0.7101 (4)	0.1074 (3)	0.0879 (18)	
H31	0.8314	0.7825	0.1055	0.105*	
C32	0.7707 (3)	0.6867 (5)	0.1229 (3)	0.097 (2)	
H32	0.7484	0.7430	0.1330	0.117*	
C33	0.7525 (2)	0.5796 (5)	0.1242 (3)	0.0843 (16)	
H33	0.7186	0.5639	0.1364	0.101*	
C34	0.78519 (19)	0.4953 (4)	0.1070 (2)	0.0644 (12)	
H34	0.7725	0.4233	0.1057	0.077*	
C35	0.87370 (19)	0.4315 (3)	0.0756 (2)	0.0495 (10)	
C36	0.87985 (16)	0.0701 (3)	0.2347 (2)	0.0441 (9)	
C37	0.83292 (18)	−0.0038 (3)	0.2274 (3)	0.0509 (10)	
C38	0.8293 (2)	−0.0506 (4)	0.2951 (3)	0.0703 (13)	
H38	0.7979	−0.0984	0.2906	0.084*	
C39	0.8722 (2)	−0.0267 (4)	0.3695 (3)	0.0769 (14)	
H39	0.8692	−0.0588	0.4150	0.092*	
C40	0.9200 (2)	0.0443 (4)	0.3788 (3)	0.0710 (13)	
H40	0.9492	0.0587	0.4294	0.085*	
C41	0.92263 (18)	0.0929 (3)	0.3104 (2)	0.0570 (11)	
H41	0.9537	0.1417	0.3153	0.068*	
C42	0.88268 (18)	0.1215 (3)	0.1610 (2)	0.0460 (9)	
C43	0.84987 (19)	0.1941 (3)	−0.1155 (2)	0.0556 (11)	
C44	0.8485 (2)	0.1465 (4)	−0.1871 (3)	0.0698 (13)	
H44	0.8120	0.1430	−0.2311	0.084*	
C45	0.8992 (2)	0.1054 (4)	−0.1936 (3)	0.0722 (13)	
H45	0.8973	0.0744	−0.2419	0.087*	
C46	0.9546 (2)	0.1095 (3)	−0.1280 (2)	0.0569 (11)	
C47	1.0111 (3)	0.0680 (3)	−0.1272 (3)	0.0724 (14)	
H47	1.0125	0.0359	−0.1735	0.087*	
C48	1.0621 (2)	0.0744 (3)	−0.0611 (3)	0.0724 (14)	
H48	1.0980	0.0468	−0.0627	0.087*	
C49	1.0621 (2)	0.1227 (3)	0.0115 (3)	0.0582 (11)	
C50	1.1135 (2)	0.1331 (3)	0.0828 (3)	0.0708 (13)	
H50	1.1506	0.1060	0.0852	0.085*	
C51	1.1092 (2)	0.1825 (4)	0.1482 (3)	0.0721 (13)	
H51	1.1435	0.1902	0.1950	0.087*	
C52	1.05307 (18)	0.2222 (3)	0.1457 (3)	0.0544 (11)	
C53	1.00751 (17)	0.1630 (3)	0.0127 (2)	0.0458 (9)	
C54	0.95339 (18)	0.1572 (3)	−0.0572 (2)	0.0472 (10)	
C55	0.7945 (2)	0.2406 (4)	−0.1093 (3)	0.0784 (14)	
H55A	0.7885	0.2097	−0.0636	0.118*	
H55B	0.7602	0.2239	−0.1574	0.118*	
H55C	0.7987	0.3183	−0.1030	0.118*	
C56	1.04876 (19)	0.2811 (4)	0.2161 (2)	0.0688 (13)	
H56A	1.0179	0.3361	0.1976	0.103*	
H56B	1.0870	0.3148	0.2463	0.103*	
H56C	1.0386	0.2303	0.2499	0.103*	

### Atomic displacement parameters (Å^2^)

**Table:**

	*U*^11^	*U*^22^	*U*^33^	*U*^12^	*U*^13^	*U*^23^
Cu1	0.0443 (3)	0.0543 (3)	0.0349 (3)	−0.0088 (2)	0.0174 (2)	−0.0012 (2)
Cu2	0.0473 (3)	0.0509 (3)	0.0462 (3)	0.0020 (2)	0.0221 (2)	−0.0010 (2)
O1	0.079 (2)	0.090 (2)	0.061 (2)	0.0205 (18)	0.0053 (18)	−0.0111 (17)
O2	0.0684 (18)	0.0526 (17)	0.0416 (15)	−0.0112 (14)	0.0223 (14)	−0.0057 (13)
O3	0.164 (4)	0.076 (2)	0.073 (2)	0.045 (3)	0.046 (2)	0.0203 (19)
O4	0.0520 (17)	0.0604 (17)	0.0450 (15)	−0.0038 (14)	0.0180 (13)	0.0105 (13)
O5	0.071 (2)	0.0692 (19)	0.0609 (18)	0.0028 (15)	0.0367 (16)	0.0050 (15)
O6	0.071 (2)	0.067 (2)	0.074 (2)	0.0095 (16)	0.0252 (18)	0.0059 (17)
O7	0.0567 (19)	0.073 (2)	0.085 (2)	−0.0001 (16)	0.0352 (17)	−0.0041 (17)
O8	0.0611 (18)	0.0504 (17)	0.073 (2)	0.0047 (14)	0.0261 (15)	−0.0054 (15)
O9	0.119 (3)	0.066 (2)	0.121 (3)	−0.025 (2)	0.048 (3)	−0.003 (2)
O10	0.0480 (16)	0.0677 (18)	0.0506 (17)	−0.0042 (14)	0.0235 (13)	0.0045 (14)
O11	0.0665 (18)	0.0551 (17)	0.0483 (17)	0.0008 (14)	0.0211 (15)	−0.0071 (13)
O12	0.066 (2)	0.0548 (19)	0.079 (2)	−0.0062 (15)	0.0244 (18)	−0.0037 (16)
N1	0.0407 (17)	0.0423 (17)	0.0376 (17)	−0.0067 (14)	0.0189 (14)	−0.0010 (14)
N2	0.0404 (18)	0.0444 (18)	0.0396 (18)	−0.0016 (14)	0.0184 (14)	0.0023 (14)
N3	0.055 (2)	0.0425 (18)	0.0437 (19)	−0.0013 (15)	0.0212 (17)	0.0019 (14)
N4	0.0475 (19)	0.0410 (18)	0.050 (2)	−0.0027 (14)	0.0208 (17)	0.0015 (15)
C1	0.053 (2)	0.047 (2)	0.055 (3)	−0.0010 (19)	0.033 (2)	−0.006 (2)
C2	0.091 (4)	0.058 (3)	0.068 (3)	0.009 (3)	0.049 (3)	0.003 (3)
C3	0.114 (5)	0.047 (3)	0.118 (5)	−0.001 (3)	0.084 (4)	0.001 (3)
C4	0.079 (4)	0.067 (3)	0.108 (4)	−0.017 (3)	0.056 (3)	−0.032 (3)
C5	0.058 (3)	0.066 (3)	0.079 (3)	−0.005 (2)	0.024 (2)	−0.020 (3)
C6	0.058 (3)	0.048 (2)	0.060 (3)	0.000 (2)	0.026 (2)	−0.008 (2)
C7	0.052 (3)	0.068 (3)	0.045 (3)	0.008 (2)	0.027 (2)	−0.004 (2)
C8	0.050 (2)	0.043 (2)	0.037 (2)	−0.0044 (18)	0.0135 (19)	−0.0027 (17)
C9	0.070 (3)	0.046 (2)	0.049 (3)	−0.001 (2)	0.021 (2)	−0.007 (2)
C10	0.082 (3)	0.072 (3)	0.047 (3)	0.014 (3)	0.009 (3)	0.006 (2)
C11	0.121 (5)	0.082 (4)	0.048 (3)	0.018 (3)	0.032 (3)	0.024 (3)
C12	0.100 (4)	0.087 (4)	0.067 (3)	0.004 (3)	0.046 (3)	0.017 (3)
C13	0.069 (3)	0.063 (3)	0.051 (3)	0.000 (2)	0.024 (2)	0.011 (2)
C14	0.056 (3)	0.043 (2)	0.041 (2)	−0.012 (2)	0.016 (2)	−0.0079 (18)
C15	0.044 (2)	0.045 (2)	0.048 (2)	−0.0004 (18)	0.0222 (19)	0.0019 (18)
C16	0.048 (2)	0.060 (3)	0.063 (3)	−0.003 (2)	0.033 (2)	0.003 (2)
C17	0.067 (3)	0.057 (3)	0.056 (3)	0.005 (2)	0.042 (2)	0.010 (2)
C18	0.056 (3)	0.044 (2)	0.043 (2)	−0.0002 (18)	0.026 (2)	−0.0003 (18)
C19	0.073 (3)	0.063 (3)	0.039 (2)	−0.002 (2)	0.026 (2)	−0.001 (2)
C20	0.068 (3)	0.069 (3)	0.040 (2)	−0.012 (2)	0.015 (2)	−0.007 (2)
C21	0.044 (2)	0.052 (2)	0.039 (2)	−0.0060 (18)	0.0098 (18)	0.0031 (18)
C22	0.047 (3)	0.068 (3)	0.049 (3)	−0.010 (2)	0.005 (2)	−0.001 (2)
C23	0.035 (2)	0.079 (3)	0.057 (3)	−0.009 (2)	0.014 (2)	0.011 (2)
C24	0.041 (2)	0.058 (3)	0.048 (2)	0.0000 (19)	0.020 (2)	0.0086 (19)
C25	0.043 (2)	0.040 (2)	0.035 (2)	−0.0033 (16)	0.0169 (18)	0.0015 (16)
C26	0.044 (2)	0.0333 (19)	0.037 (2)	0.0037 (16)	0.0184 (18)	0.0030 (16)
C27	0.042 (2)	0.085 (3)	0.062 (3)	−0.013 (2)	0.020 (2)	−0.006 (2)
C28	0.050 (3)	0.097 (3)	0.060 (3)	−0.005 (2)	0.032 (2)	0.004 (2)
C29	0.052 (3)	0.044 (2)	0.038 (2)	0.0078 (19)	0.0053 (19)	−0.0018 (18)
C30	0.070 (3)	0.054 (3)	0.051 (3)	0.003 (2)	0.010 (2)	−0.004 (2)
C31	0.105 (4)	0.053 (3)	0.076 (4)	0.026 (3)	0.000 (3)	−0.013 (3)
C32	0.092 (4)	0.092 (5)	0.082 (4)	0.051 (4)	0.003 (3)	−0.019 (3)
C33	0.055 (3)	0.117 (5)	0.073 (3)	0.030 (3)	0.014 (3)	−0.005 (3)
C34	0.054 (3)	0.076 (3)	0.060 (3)	0.013 (2)	0.018 (2)	0.000 (2)
C35	0.054 (3)	0.053 (3)	0.039 (2)	0.006 (2)	0.015 (2)	0.0022 (19)
C36	0.046 (2)	0.043 (2)	0.049 (2)	0.0101 (18)	0.024 (2)	0.0017 (18)
C37	0.055 (3)	0.045 (2)	0.063 (3)	0.011 (2)	0.034 (2)	0.005 (2)
C38	0.079 (3)	0.061 (3)	0.088 (4)	0.005 (2)	0.052 (3)	0.010 (3)
C39	0.107 (4)	0.071 (3)	0.078 (4)	0.017 (3)	0.064 (3)	0.016 (3)
C40	0.086 (4)	0.078 (3)	0.051 (3)	0.016 (3)	0.027 (3)	0.006 (2)
C41	0.064 (3)	0.057 (3)	0.054 (3)	0.007 (2)	0.027 (2)	0.000 (2)
C42	0.049 (2)	0.039 (2)	0.057 (3)	0.0128 (19)	0.028 (2)	−0.0004 (19)
C43	0.063 (3)	0.056 (3)	0.043 (2)	0.000 (2)	0.015 (2)	0.003 (2)
C44	0.087 (4)	0.070 (3)	0.044 (3)	0.000 (3)	0.016 (3)	−0.005 (2)
C45	0.115 (4)	0.064 (3)	0.042 (3)	0.000 (3)	0.033 (3)	−0.006 (2)
C46	0.090 (3)	0.042 (2)	0.053 (3)	−0.001 (2)	0.043 (3)	−0.001 (2)
C47	0.109 (4)	0.050 (3)	0.088 (4)	0.004 (3)	0.071 (3)	−0.001 (3)
C48	0.080 (4)	0.051 (3)	0.116 (4)	0.002 (2)	0.071 (3)	−0.002 (3)
C49	0.067 (3)	0.037 (2)	0.087 (3)	0.001 (2)	0.049 (3)	0.001 (2)
C50	0.051 (3)	0.055 (3)	0.114 (4)	0.005 (2)	0.040 (3)	0.008 (3)
C51	0.053 (3)	0.060 (3)	0.092 (4)	−0.004 (2)	0.014 (3)	0.002 (3)
C52	0.047 (3)	0.051 (2)	0.063 (3)	−0.006 (2)	0.017 (2)	0.004 (2)
C53	0.056 (3)	0.032 (2)	0.060 (3)	−0.0012 (18)	0.035 (2)	0.0052 (18)
C54	0.062 (3)	0.039 (2)	0.049 (2)	−0.0037 (19)	0.030 (2)	0.0029 (18)
C55	0.056 (3)	0.101 (4)	0.064 (3)	0.003 (3)	0.006 (2)	−0.010 (3)
C56	0.060 (3)	0.080 (3)	0.060 (3)	−0.017 (2)	0.015 (2)	−0.011 (2)

### Geometric parameters (Å, °)

**Table:**

Cu1—O2	1.931 (2)	C20—C21	1.436 (5)
Cu1—O4	1.946 (2)	C20—H20	0.9300
Cu1—N2	1.994 (3)	C21—C25	1.390 (5)
Cu1—N1	2.022 (3)	C21—C22	1.396 (5)
Cu2—O10	1.945 (2)	C22—C23	1.363 (5)
Cu2—O8	1.956 (3)	C22—H22	0.9300
Cu2—N4	1.992 (3)	C23—C24	1.405 (5)
Cu2—N3	2.044 (3)	C23—H23	0.9300
O1—C7	1.249 (4)	C24—C28	1.501 (5)
O2—C7	1.267 (4)	C25—C26	1.431 (5)
O3—C2	1.350 (5)	C27—H27A	0.9600
O3—H3	0.8200	C27—H27B	0.9600
O4—C14	1.264 (4)	C27—H27C	0.9600
O5—C14	1.254 (4)	C28—H28A	0.9600
O6—C9	1.355 (5)	C28—H28B	0.9600
O6—H6	0.8200	C28—H28C	0.9600
O7—C35	1.245 (4)	C29—C30	1.387 (6)
O8—C35	1.264 (4)	C29—C34	1.395 (5)
O9—C30	1.351 (5)	C29—C35	1.487 (5)
O9—H9	0.8200	C30—C31	1.377 (6)
O10—C42	1.275 (4)	C31—C32	1.344 (7)
O11—C42	1.254 (4)	C31—H31	0.9300
O12—C37	1.343 (4)	C32—C33	1.386 (8)
O12—H12	0.8200	C32—H32	0.9300
N1—C15	1.332 (4)	C33—C34	1.396 (6)
N1—C26	1.360 (4)	C33—H33	0.9300
N2—C24	1.332 (4)	C34—H34	0.9300
N2—C25	1.369 (4)	C36—C41	1.384 (5)
N3—C43	1.339 (5)	C36—C37	1.407 (5)
N3—C54	1.367 (4)	C36—C42	1.487 (5)
N4—C52	1.324 (5)	C37—C38	1.372 (5)
N4—C53	1.367 (4)	C38—C39	1.372 (6)
C1—C2	1.392 (5)	C38—H38	0.9300
C1—C6	1.394 (5)	C39—C40	1.393 (6)
C1—C7	1.490 (5)	C39—H39	0.9300
C2—C3	1.394 (6)	C40—C41	1.384 (5)
C3—C4	1.354 (6)	C40—H40	0.9300
C3—H3A	0.9300	C41—H41	0.9300
C4—C5	1.375 (6)	C43—C44	1.397 (5)
C4—H4	0.9300	C43—C55	1.479 (6)
C5—C6	1.373 (5)	C44—C45	1.355 (6)
C5—H5	0.9300	C44—H44	0.9300
C6—H6A	0.9300	C45—C46	1.400 (6)
C8—C13	1.381 (5)	C45—H45	0.9300
C8—C9	1.399 (5)	C46—C54	1.407 (5)
C8—C14	1.484 (5)	C46—C47	1.434 (6)
C9—C10	1.392 (6)	C47—C48	1.343 (6)
C10—C11	1.364 (6)	C47—H47	0.9300
C10—H10	0.9300	C48—C49	1.429 (6)
C11—C12	1.373 (6)	C48—H48	0.9300
C11—H11	0.9300	C49—C53	1.399 (5)
C12—C13	1.391 (5)	C49—C50	1.404 (6)
C12—H12A	0.9300	C50—C51	1.356 (6)
C13—H13	0.9300	C50—H50	0.9300
C15—C16	1.396 (5)	C51—C52	1.407 (6)
C15—C27	1.504 (5)	C51—H51	0.9300
C16—C17	1.350 (5)	C52—C56	1.490 (6)
C16—H16	0.9300	C53—C54	1.423 (5)
C17—C18	1.408 (5)	C55—H55A	0.9600
C17—H17	0.9300	C55—H55B	0.9600
C18—C26	1.399 (5)	C55—H55C	0.9600
C18—C19	1.433 (5)	C56—H56A	0.9600
C19—C20	1.346 (5)	C56—H56B	0.9600
C19—H19	0.9300	C56—H56C	0.9600
			
O2—Cu1—O4	91.00 (11)	C21—C25—C26	120.5 (3)
O2—Cu1—N2	152.67 (12)	N1—C26—C18	123.0 (3)
O4—Cu1—N2	97.15 (11)	N1—C26—C25	117.4 (3)
O2—Cu1—N1	104.90 (11)	C18—C26—C25	119.6 (3)
O4—Cu1—N1	144.27 (11)	C15—C27—H27A	109.5
N2—Cu1—N1	83.30 (11)	C15—C27—H27B	109.5
O10—Cu2—O8	90.72 (11)	H27A—C27—H27B	109.5
O10—Cu2—N4	94.59 (12)	C15—C27—H27C	109.5
O8—Cu2—N4	155.88 (12)	H27A—C27—H27C	109.5
O10—Cu2—N3	143.61 (12)	H27B—C27—H27C	109.5
O8—Cu2—N3	106.20 (12)	C24—C28—H28A	109.5
N4—Cu2—N3	82.98 (13)	C24—C28—H28B	109.5
C7—O2—Cu1	110.6 (2)	H28A—C28—H28B	109.5
C2—O3—H3	109.5	C24—C28—H28C	109.5
C14—O4—Cu1	108.9 (2)	H28A—C28—H28C	109.5
C9—O6—H6	109.5	H28B—C28—H28C	109.5
C35—O8—Cu2	104.0 (3)	C30—C29—C34	119.2 (4)
C30—O9—H9	109.5	C30—C29—C35	120.4 (4)
C42—O10—Cu2	109.0 (2)	C34—C29—C35	120.4 (4)
C37—O12—H12	109.5	O9—C30—C31	118.9 (5)
C15—N1—C26	118.7 (3)	O9—C30—C29	120.9 (4)
C15—N1—Cu1	130.4 (2)	C31—C30—C29	120.3 (5)
C26—N1—Cu1	110.8 (2)	C32—C31—C30	120.8 (5)
C24—N2—C25	118.9 (3)	C32—C31—H31	119.6
C24—N2—Cu1	129.3 (3)	C30—C31—H31	119.6
C25—N2—Cu1	111.8 (2)	C31—C32—C33	120.7 (5)
C43—N3—C54	119.1 (3)	C31—C32—H32	119.6
C43—N3—Cu2	130.4 (3)	C33—C32—H32	119.6
C54—N3—Cu2	110.1 (2)	C32—C33—C34	119.5 (5)
C52—N4—C53	120.1 (3)	C32—C33—H33	120.2
C52—N4—Cu2	128.1 (3)	C34—C33—H33	120.2
C53—N4—Cu2	111.7 (2)	C29—C34—C33	119.4 (5)
C2—C1—C6	118.3 (4)	C29—C34—H34	120.3
C2—C1—C7	121.2 (4)	C33—C34—H34	120.3
C6—C1—C7	120.5 (4)	O7—C35—O8	121.5 (4)
O3—C2—C1	121.0 (4)	O7—C35—C29	120.5 (4)
O3—C2—C3	119.5 (5)	O8—C35—C29	118.0 (4)
C1—C2—C3	119.5 (5)	C41—C36—C37	119.2 (4)
C4—C3—C2	120.5 (5)	C41—C36—C42	121.5 (4)
C4—C3—H3A	119.8	C37—C36—C42	119.3 (4)
C2—C3—H3A	119.8	O12—C37—C38	117.3 (4)
C3—C4—C5	121.1 (5)	O12—C37—C36	122.8 (4)
C3—C4—H4	119.5	C38—C37—C36	119.9 (4)
C5—C4—H4	119.5	C39—C38—C37	119.9 (5)
C6—C5—C4	119.1 (5)	C39—C38—H38	120.1
C6—C5—H5	120.5	C37—C38—H38	120.1
C4—C5—H5	120.5	C38—C39—C40	121.7 (4)
C5—C6—C1	121.5 (4)	C38—C39—H39	119.1
C5—C6—H6A	119.3	C40—C39—H39	119.1
C1—C6—H6A	119.3	C41—C40—C39	118.0 (4)
O1—C7—O2	122.3 (4)	C41—C40—H40	121.0
O1—C7—C1	119.5 (4)	C39—C40—H40	121.0
O2—C7—C1	118.2 (4)	C40—C41—C36	121.2 (4)
C13—C8—C9	118.6 (4)	C40—C41—H41	119.4
C13—C8—C14	120.9 (4)	C36—C41—H41	119.4
C9—C8—C14	120.4 (4)	O11—C42—O10	122.4 (4)
O6—C9—C10	118.0 (4)	O11—C42—C36	120.1 (4)
O6—C9—C8	122.2 (4)	O10—C42—C36	117.6 (4)
C10—C9—C8	119.8 (4)	N3—C43—C44	120.1 (4)
C11—C10—C9	120.5 (4)	N3—C43—C55	119.0 (4)
C11—C10—H10	119.7	C44—C43—C55	120.9 (4)
C9—C10—H10	119.7	C45—C44—C43	121.3 (4)
C10—C11—C12	120.5 (4)	C45—C44—H44	119.3
C10—C11—H11	119.7	C43—C44—H44	119.3
C12—C11—H11	119.7	C44—C45—C46	120.3 (4)
C11—C12—C13	119.6 (5)	C44—C45—H45	119.9
C11—C12—H12A	120.2	C46—C45—H45	119.9
C13—C12—H12A	120.2	C45—C46—C54	116.2 (4)
C8—C13—C12	121.0 (4)	C45—C46—C47	125.5 (4)
C8—C13—H13	119.5	C54—C46—C47	118.3 (4)
C12—C13—H13	119.5	C48—C47—C46	121.6 (4)
O5—C14—O4	122.8 (4)	C48—C47—H47	119.2
O5—C14—C8	119.8 (4)	C46—C47—H47	119.2
O4—C14—C8	117.4 (4)	C47—C48—C49	121.2 (4)
N1—C15—C16	121.0 (3)	C47—C48—H48	119.4
N1—C15—C27	118.5 (3)	C49—C48—H48	119.4
C16—C15—C27	120.4 (3)	C53—C49—C50	116.9 (4)
C17—C16—C15	121.0 (4)	C53—C49—C48	118.4 (4)
C17—C16—H16	119.5	C50—C49—C48	124.7 (4)
C15—C16—H16	119.5	C51—C50—C49	120.2 (4)
C16—C17—C18	119.5 (4)	C51—C50—H50	119.9
C16—C17—H17	120.3	C49—C50—H50	119.9
C18—C17—H17	120.3	C50—C51—C52	120.3 (4)
C26—C18—C17	116.9 (3)	C50—C51—H51	119.8
C26—C18—C19	118.7 (4)	C52—C51—H51	119.8
C17—C18—C19	124.5 (4)	N4—C52—C51	120.3 (4)
C20—C19—C18	121.7 (4)	N4—C52—C56	119.3 (4)
C20—C19—H19	119.2	C51—C52—C56	120.4 (4)
C18—C19—H19	119.2	N4—C53—C49	122.0 (4)
C19—C20—C21	120.5 (4)	N4—C53—C54	117.3 (3)
C19—C20—H20	119.8	C49—C53—C54	120.7 (4)
C21—C20—H20	119.8	N3—C54—C46	123.1 (4)
C25—C21—C22	117.4 (4)	N3—C54—C53	117.2 (3)
C25—C21—C20	119.0 (3)	C46—C54—C53	119.7 (4)
C22—C21—C20	123.6 (4)	C43—C55—H55A	109.5
C23—C22—C21	119.3 (4)	C43—C55—H55B	109.5
C23—C22—H22	120.3	H55A—C55—H55B	109.5
C21—C22—H22	120.3	C43—C55—H55C	109.5
C22—C23—C24	121.0 (4)	H55A—C55—H55C	109.5
C22—C23—H23	119.5	H55B—C55—H55C	109.5
C24—C23—H23	119.5	C52—C56—H56A	109.5
N2—C24—C23	120.2 (4)	C52—C56—H56B	109.5
N2—C24—C28	119.0 (3)	H56A—C56—H56B	109.5
C23—C24—C28	120.7 (3)	C52—C56—H56C	109.5
N2—C25—C21	123.0 (3)	H56A—C56—H56C	109.5
N2—C25—C26	116.6 (3)	H56B—C56—H56C	109.5
			
O4—Cu1—O2—C7	141.8 (3)	C24—N2—C25—C26	−176.5 (3)
N2—Cu1—O2—C7	34.1 (4)	Cu1—N2—C25—C26	4.6 (4)
N1—Cu1—O2—C7	−70.5 (3)	C22—C21—C25—N2	0.2 (5)
O2—Cu1—O4—C14	83.0 (2)	C20—C21—C25—N2	−178.6 (3)
N2—Cu1—O4—C14	−123.1 (2)	C22—C21—C25—C26	−179.8 (3)
N1—Cu1—O4—C14	−34.7 (3)	C20—C21—C25—C26	1.4 (5)
O10—Cu2—O8—C35	104.0 (2)	C15—N1—C26—C18	0.5 (5)
N4—Cu2—O8—C35	1.0 (4)	Cu1—N1—C26—C18	177.7 (3)
N3—Cu2—O8—C35	−108.7 (2)	C15—N1—C26—C25	−177.9 (3)
O8—Cu2—O10—C42	75.4 (2)	Cu1—N1—C26—C25	−0.7 (4)
N4—Cu2—O10—C42	−128.2 (2)	C17—C18—C26—N1	1.5 (5)
N3—Cu2—O10—C42	−43.8 (3)	C19—C18—C26—N1	−178.9 (3)
O2—Cu1—N1—C15	−27.3 (3)	C17—C18—C26—C25	179.8 (3)
O4—Cu1—N1—C15	86.4 (3)	C19—C18—C26—C25	−0.6 (5)
N2—Cu1—N1—C15	179.3 (3)	N2—C25—C26—N1	−2.7 (5)
O2—Cu1—N1—C26	155.9 (2)	C21—C25—C26—N1	177.4 (3)
O4—Cu1—N1—C26	−90.4 (3)	N2—C25—C26—C18	178.9 (3)
N2—Cu1—N1—C26	2.5 (2)	C21—C25—C26—C18	−1.1 (5)
O2—Cu1—N2—C24	67.7 (4)	C34—C29—C30—O9	176.9 (4)
O4—Cu1—N2—C24	−38.6 (3)	C35—C29—C30—O9	−1.1 (6)
N1—Cu1—N2—C24	177.4 (3)	C34—C29—C30—C31	−3.3 (6)
O2—Cu1—N2—C25	−113.6 (3)	C35—C29—C30—C31	178.7 (4)
O4—Cu1—N2—C25	140.1 (2)	O9—C30—C31—C32	−176.1 (5)
N1—Cu1—N2—C25	−3.9 (2)	C29—C30—C31—C32	4.0 (7)
O10—Cu2—N3—C43	91.7 (4)	C30—C31—C32—C33	−1.5 (8)
O8—Cu2—N3—C43	−22.9 (4)	C31—C32—C33—C34	−1.6 (8)
N4—Cu2—N3—C43	179.9 (3)	C30—C29—C34—C33	0.2 (6)
O10—Cu2—N3—C54	−81.1 (3)	C35—C29—C34—C33	178.1 (4)
O8—Cu2—N3—C54	164.3 (2)	C32—C33—C34—C29	2.3 (7)
N4—Cu2—N3—C54	7.1 (2)	Cu2—O8—C35—O7	8.9 (4)
O10—Cu2—N4—C52	−40.7 (3)	Cu2—O8—C35—C29	−169.2 (3)
O8—Cu2—N4—C52	61.4 (5)	C30—C29—C35—O7	13.4 (6)
N3—Cu2—N4—C52	175.8 (3)	C34—C29—C35—O7	−164.5 (4)
O10—Cu2—N4—C53	136.2 (2)	C30—C29—C35—O8	−168.4 (4)
O8—Cu2—N4—C53	−121.6 (3)	C34—C29—C35—O8	13.7 (5)
N3—Cu2—N4—C53	−7.3 (2)	C41—C36—C37—O12	−178.0 (3)
C6—C1—C2—O3	177.9 (4)	C42—C36—C37—O12	1.6 (5)
C7—C1—C2—O3	−0.8 (6)	C41—C36—C37—C38	1.4 (5)
C6—C1—C2—C3	−2.7 (6)	C42—C36—C37—C38	−179.0 (3)
C7—C1—C2—C3	178.7 (4)	O12—C37—C38—C39	178.1 (4)
O3—C2—C3—C4	−177.4 (5)	C36—C37—C38—C39	−1.3 (6)
C1—C2—C3—C4	3.2 (7)	C37—C38—C39—C40	−0.1 (7)
C2—C3—C4—C5	−1.7 (7)	C38—C39—C40—C41	1.4 (7)
C3—C4—C5—C6	−0.3 (7)	C39—C40—C41—C36	−1.3 (6)
C4—C5—C6—C1	0.7 (6)	C37—C36—C41—C40	−0.1 (6)
C2—C1—C6—C5	0.7 (6)	C42—C36—C41—C40	−179.6 (4)
C7—C1—C6—C5	179.4 (4)	Cu2—O10—C42—O11	0.7 (4)
Cu1—O2—C7—O1	−3.1 (5)	Cu2—O10—C42—C36	−179.8 (2)
Cu1—O2—C7—C1	175.1 (3)	C41—C36—C42—O11	178.5 (3)
C2—C1—C7—O1	5.6 (6)	C37—C36—C42—O11	−1.1 (5)
C6—C1—C7—O1	−173.1 (4)	C41—C36—C42—O10	−1.0 (5)
C2—C1—C7—O2	−172.7 (4)	C37—C36—C42—O10	179.4 (3)
C6—C1—C7—O2	8.7 (5)	C54—N3—C43—C44	0.8 (6)
C13—C8—C9—O6	−178.6 (4)	Cu2—N3—C43—C44	−171.4 (3)
C14—C8—C9—O6	4.4 (5)	C54—N3—C43—C55	−178.9 (4)
C13—C8—C9—C10	1.6 (6)	Cu2—N3—C43—C55	8.8 (6)
C14—C8—C9—C10	−175.5 (3)	N3—C43—C44—C45	−0.8 (7)
O6—C9—C10—C11	179.4 (4)	C55—C43—C44—C45	178.9 (4)
C8—C9—C10—C11	−0.8 (6)	C43—C44—C45—C46	0.3 (7)
C9—C10—C11—C12	−0.7 (8)	C44—C45—C46—C54	0.3 (6)
C10—C11—C12—C13	1.4 (8)	C44—C45—C46—C47	179.4 (4)
C9—C8—C13—C12	−1.0 (6)	C45—C46—C47—C48	−179.4 (4)
C14—C8—C13—C12	176.1 (4)	C54—C46—C47—C48	−0.3 (6)
C11—C12—C13—C8	−0.5 (7)	C46—C47—C48—C49	0.2 (7)
Cu1—O4—C14—O5	9.1 (4)	C47—C48—C49—C53	0.4 (6)
Cu1—O4—C14—C8	−170.0 (2)	C47—C48—C49—C50	−179.6 (4)
C13—C8—C14—O5	173.0 (4)	C53—C49—C50—C51	−1.4 (6)
C9—C8—C14—O5	−9.9 (5)	C48—C49—C50—C51	178.5 (4)
C13—C8—C14—O4	−7.8 (5)	C49—C50—C51—C52	1.0 (7)
C9—C8—C14—O4	169.2 (3)	C53—N4—C52—C51	−3.6 (6)
C26—N1—C15—C16	−1.6 (5)	Cu2—N4—C52—C51	173.1 (3)
Cu1—N1—C15—C16	−178.2 (3)	C53—N4—C52—C56	175.1 (3)
C26—N1—C15—C27	176.9 (3)	Cu2—N4—C52—C56	−8.2 (5)
Cu1—N1—C15—C27	0.3 (5)	C50—C51—C52—N4	1.6 (6)
N1—C15—C16—C17	0.7 (6)	C50—C51—C52—C56	−177.2 (4)
C27—C15—C16—C17	−177.7 (4)	C52—N4—C53—C49	3.2 (5)
C15—C16—C17—C18	1.3 (6)	Cu2—N4—C53—C49	−174.0 (3)
C16—C17—C18—C26	−2.3 (5)	C52—N4—C53—C54	−176.5 (3)
C16—C17—C18—C19	178.1 (4)	Cu2—N4—C53—C54	6.2 (4)
C26—C18—C19—C20	2.0 (6)	C50—C49—C53—N4	−0.6 (5)
C17—C18—C19—C20	−178.4 (4)	C48—C49—C53—N4	179.4 (3)
C18—C19—C20—C21	−1.7 (6)	C50—C49—C53—C54	179.1 (3)
C19—C20—C21—C25	0.0 (6)	C48—C49—C53—C54	−0.9 (5)
C19—C20—C21—C22	−178.7 (4)	C43—N3—C54—C46	−0.2 (5)
C25—C21—C22—C23	−2.4 (6)	Cu2—N3—C54—C46	173.5 (3)
C20—C21—C22—C23	176.3 (4)	C43—N3—C54—C53	−179.6 (3)
C21—C22—C23—C24	1.1 (6)	Cu2—N3—C54—C53	−5.9 (4)
C25—N2—C24—C23	−4.8 (5)	C45—C46—C54—N3	−0.4 (5)
Cu1—N2—C24—C23	173.8 (3)	C47—C46—C54—N3	−179.5 (3)
C25—N2—C24—C28	174.4 (3)	C45—C46—C54—C53	179.0 (4)
Cu1—N2—C24—C28	−7.0 (5)	C47—C46—C54—C53	−0.2 (5)
C22—C23—C24—N2	2.6 (6)	N4—C53—C54—N3	−0.1 (5)
C22—C23—C24—C28	−176.5 (4)	C49—C53—C54—N3	−179.9 (3)
C24—N2—C25—C21	3.4 (5)	N4—C53—C54—C46	−179.5 (3)
Cu1—N2—C25—C21	−175.4 (3)	C49—C53—C54—C46	0.7 (5)

### Hydrogen-bond geometry (Å, °)

**Table:**

*D*—H···*A*	*D*—H	H···*A*	*D*···*A*	*D*—H···*A*
O12—H12···O11	0.82	1.82	2.549 (4)	147
O9—H9···O7	0.82	1.84	2.561 (4)	146
O6—H6···O5	0.82	1.85	2.572 (4)	146
O3—H3···O1	0.82	1.82	2.553 (5)	148
